# A diagnosis of chronic kidney disease: despite fears patients want to know early 

**DOI:** 10.5414/CN108831

**Published:** 2016-06-27

**Authors:** Julie Wright Nunes, Meghan Roney, Eve Kerr, Akinlolu Ojo, Angela Fagerlin

**Affiliations:** 1 Internal Medicine, University of Michigan,; 2 Center for Bioethics and Social Sciences in Medicine, and; 3 Veterans Affairs Ann Arbor Center for Clinical Management Research, Ann Arbor, MI, USA

**Keywords:** patient education, chronic kidney disease, interviews, quality improvement

## Abstract

Aims: We elicited input from patients on their experience getting a chronic kidney disease (CKD) diagnosis to use for optimizing current CKD education interventions. Methods: We performed structured one-on-one patient interviews. Interviews were recorded, transcribed, and coded using modified grounded theory. Participants had CKD, were not on dialysis, and were recruited from general nephrology practices. Results: 49 patients enrolled from January to October 2014. Interviews revealed four major themes: 1. Reaction to diagnosis – patients described emotional reactions and subsequent behavior changes (152 statements); 2. Timing of diagnosis – patients described how they were told about their diagnosis and expectations of when a person should be told (149 statements); 3. Mediators in diagnosis delivery – patients discussed things that helped or hindered understanding and acceptance of their diagnosis (64 statements), and 4. Perceptions of diagnosis terminology – patients discussed perceptions about diagnostic terms (e.g., “chronic kidney disease”) (91 statements). Cross-sectional study design and setting limit interpretation of causality and generalizability. Conclusions: Patients experience fear but prefer early diagnosis communication. More work is needed to define evidence-based guidelines for diagnosis messaging across the spectrum of care.

Supplemental material is available for free at: http://www.clinnephrol.com Vol. 86 – August 2016

## Introduction 

Most patients with chronic kidney disease (CKD) are unaware of their diagnosis [1]. Those who are aware are often ill-informed about its meaning and what they can do to preserve existing kidney function [2]. Low patient awareness and knowledge about CKD are likely due to multiple factors. Research links low patient awareness with low socio-economic status, less formal education, and low health literacy [2, 3]. Qualitative research also suggests there are barriers at the provider level [4]. 

Focus group research with primary care providers reveals they have concerns about telling patients they have CKD [4]. They are concerned it can create unnecessary stress for patients, especially if patient awareness will not change how the clinician believes the patient’s care will be managed. Limited data from patient focus groups indicate patients want more diagnosis information including information on disease management and preparation for renal replacement therapies [5]. 

The chronic care model put forth by the National Academy of Medicine (formerly the Institute of Medicine) emphasizes patients at the center of care [6]. National policies and programs promote this model for disease screening and education [7]. But there is a paucity of literature describing perspectives from patients on how it is best to learn about their diagnosis and ways to optimize messaging during diagnosis delivery. Without this information we are missing important insights from those most central to CKD care in how to facilitate disease education starting with telling a person they have a disease. 

We designed a study to get input from patients on how to improve their experience receiving a diagnosis of CKD. Quality function deployment and cause/effect analysis are two quality improvement (QI) methods used in the corporate sector to systematically collect perspectives from the “voice of the customer” [8, 9, 10]. We used these methodologies to develop questions for semi-structured interviews of patients. We asked patients how and whether early diagnosis information supports overall care and how diagnosis messaging could be optimized. We planned to use this information for developing educational tools to optimize kidney disease education across the spectrum of care. 

## Methods 

### Research team 

Members of the research team have backgrounds in qualitative and quantitative research, quality improvement, medicine, psychology, public health, decision science, and health services research. Interviews were conducted by four members of the study team. 

### Study design 

This research is part of a qualitative study seeking information about patient needs and factors that influence CKD self-care and health-related decision making. An interview guide was developed by the research team, with topics informed by prior work – with particular focus on questions not yet well-elucidated [5, 11, 12, 13, 14]. Content was refined for an interview length of ~ 30 minutes. 

Interview questions were developed using principles of cause/effect analysis and quality function deployment (see Supplemental material for specific interview questions pertaining to this study). “Cause/effect” analysis has been used by corporate entities and manufacturers to analyze and solve problems related to low production, quality defects, and employee injury rates. Through iterative steps seemingly large and insurmountable problems are broken down into smaller causal components that can be defined to eliminate their overall negative effect [8]. “Quality function deployment” (QFD) utilizes a systematic approach of rank-ordering attributes, preferences, and desires to guide a development process. QFD promotes common understanding of stakeholder needs and guides development to create best possible end-products [9, 10]. We used these methods as a framework around which the interview questions were designed (Table 1). 

### Participants and setting 

Patients with CKD were enrolled from several outpatient nephrology clinics within one academic medical center. Interviews occurred in private rooms around clinics. Inclusion criteria were: 1) age ≥ 18 years, 2) CKD stages 1 – 5 [15], 3) ability to understand/speak English, and 4) established patient. Patients receiving dialysis, with a renal transplant, or severe cognitive impairment were excluded. Written informed consent was obtained from all participants. The Institutional Review Board at the University of Michigan approved all study procedures prior to enrollment. 

### Data collection 

Measured characteristics include age, sex, race, ethnicity, and CKD stage. Interviews were audio-recorded and transcribed verbatim. The first 10 participants were asked a few additional questions to get feedback on question clarity. We planned to enroll until additional interviews did not provide new information (thematic saturation). 

### Data analysis 

Transcripts were imported into Dedoose^TM^, a qualitative data analysis package. The framework for analysis was developed using the concept of modified grounded theory [16, 17] involving cyclical and iterative steps where data elements are coded and categorized as they emerge [18]. We used the theoretical framework of a modified grounded theory for synthesis because it enabled us to seek out and conceptualize themes based upon participant responses, using a systematically applied set of methods. This framework emphasizes allowing theories to emerge from data that is gathered instead of attempting to collect information in order to “prove” prior speculations. The idea is that theory derived from data reflects reality more than speculation [16]. Modified grounded theory framework builds rather than tests theories and promotes systematic methods to handle interpretation of large amounts of raw data or text. An initial coding framework was anticipated based on qualitative work in other populations [19, 20, 21, 22] and then further developed by the research team, informed by the interviews. 

Initially, we drafted major categories of themes from interview responses to each interview question (e.g., barriers to understanding a CKD diagnosis, perceptions of patient diagnosis awareness) (please see Supplemental material for questions). After we completed the interviews, two study team members reviewed each interview transcript in detail separately and made notes about how to revise draft categories of themes to reflect all participant responses brought forth. Two investigators discussed and revised themes to ensure they reflected input until a final coding schema emerged. Using the final coding schema, two investigators re-reviewed each transcript individually, applying lines of text to each theme. The investigators met to discuss individually coded applications weekly, confirm agreement of the applied codes, and resolve differences by consensus, which at times included getting input from a third study team member. We created a third and final consensus document for every transcript based on the final agreed upon themes and code applications and used it in the final analyses. Descriptive statistics from a summary of the consensus documents are reported in frequency and percentages. Within each theme patients at times provided more granular information, which we included in the results description section for each major theme. 

## Results 

Patients were enrolled from January to October 2014. Thematic saturation arose after interviewing 47 patients but we continued through 50 participants to confirm additional themes. One audio-file was invalidated because of recorder malfunction, and not included in the final analysis. 

The mean (SD) age of participants was 62 (14) years, 51% were women, and 81% white. 24 (49%) said they did not know what caused their kidney disease. Characteristics are reported in Table 2. 

Four major themes emerged about the patient experience of getting a CKD diagnosis: 1. Reaction to diagnosis, 2. Timing of diagnosis, 3. Mediators in diagnosis delivery, and 4. Perceptions of diagnosis terminology (e.g., “chronic kidney disease”). Below are descriptions of each theme with representative statements. Some statements overlapped themes. “Non-contributory” statements (patient discussing unrelated information or off topic) are not included. 

### Reaction to diagnosis (152 total statements) 

The majority of statements here focused on emotions after getting a CKD diagnosis. Most statements reflected fear of the “unknown” and of dialysis. Additional comments relayed disbelief and even frustration that diagnosis messaging had not occurred earlier. Few comments were about behavior changes. Patient descriptors are CKD stage, age range in years, race, and sex. 

**Emotions**

- “Well I was … concerned, not confused, but I was concerned about when and if ever I’d have to go on dialysis.” CKD 3, 70+ years, female, white
- “I just feel like it’s got me controlled and I … don’t want to end up in dialysis. So that’s about kidney disease the most frightening thing of all is dialysis. Because I have 4 friends on dialysis plus my sister died, and I think dialysis killed her, and my friends none of them out of the 4 do well on dialysis.“ CKD 3, 70+ years, male, white
- “Well, you know, you get emotional feelings … Pressure on you … you don’t know what’s going to happen. This could turn into a whole bunch of other things.” CKD 4, 70+ years, female, white
- “Um, most confusing is, if I’ve … had this problem, why did no one tell me years ago? Basically is not being notified or there was not enough communication as far as health providers or doctors to let me know about my own health so I could prevent some things from happening.” CKD 5, 30 – 50 years, male, black

**Behavior changes:**

- “Well, I definitely try to follow all medical instructions. I watched the different medications that I was taking, particularly because some effect the kidneys and dramatically. I don’t think in terms of exercise or diet I made changes immediately, but I’d say maybe within the last 10 years or so I definitely have been more ... uh … paid more attention ...” CKD 5, 50 – 70 years, male, white
- “I’ve since changed my diet …” CKD 3, 30 – 50 years, male, white
- “I used to take ibuprofen for my arthritis, but NSAIDs, ibuprofen is an NSAID, and they don’t recommend people with chronic kidney disease to take NSAIDs … So I have stopped taking that and I take Tylenol and some other things instead.” CKD 3, 50 – 70 years, female, white
- “Nah, I haven’t done … haven’t really changed … No.” CKD 4, 50 – 70 years, female, multi-race

### Timing of diagnosis (149 total statements) 

Statements here described how patients heard about their diagnosis and when in the spectrum of disease patients felt they should be informed. The majority said they wanted to know about their CKD diagnosis as soon as a “problem” was identified.

- “Yes, actually the first valid alert was when I got refused for an insurance policy (laughs) and they sent me a letter saying this is why, and I immediately called the doctor because I hadn’t heard that diagnosis before … chronic kidney disease I hadn’t heard it …” CKD 3, 50 – 70 years, male, white
- “I think they (patients) should be told very early on. It’s like saying I’m gonna protect the patient and then now he’s really bad off. I don’t think that’s a very good philosophy …” CKD 2, 50 – 70 years, male, white
- “They should be a straight shooter. Let them know off the bat because if you don’t tell me, you know, upfront … So just be upfront, you know, and give the information right then so they know what direction to go.” CKD 3, 50 – 70 years, female, black
- “I think it … it really depends on how the discussion is delivered... So I think a lot of that, um … if … if you explain it reasonably well in … in terms of … of what the significance, particularly regarding mortality is concerned, you can probably do it at an earlier stage and not freak the patient out.” CKD 2, 50 – 70, female, Asian
- “Well with polycystic kidneys … uh … I think the sooner that you know you have it the better …” CKD 5, 50 – 70 years, male, white
- “I have always been a firm believer of honesty is always the best policy, and the doctor I believe should tell them but be important in emphasizing this is just the beginning, there is no reason for you to stress out over it, we will be monitoring you in the years to come …” CKD 4, 70+ years, female, white

### Mediators in diagnosis delivery (64 total statements) 

Here patients described things that made it hard to accept and/or understand their diagnosis. Most statements focused on barriers to understanding with few related to facilitators. 

**Barriers to understanding diagnosis:**

- “… the biggest thing is I was sick… I didn’t feel good, so (even) though my doctor was, was describing it for me and explaining and stuff, I don’t know if it was going in because there were so many other things, you know, wrong …” CKD 4, 50 – 70 years, male, white
- “The other part about all of this is that there isn’t much you can do, if there is anything you can do, to change the way the disease is going to go … You can change your diet and there is the usual stuff but there is. But in some diseases if you cut out cholesterol and take Crestor, boy, that (laughs), you know that’s a big help ... In retrospect, there is nothing (for CKD) ...” CKD 3, 70+, male, white
- “Well, I think (pause) the biggest barrier is … that if you don’t have any real symptomology other than maybe your legs are swollen ... you don’t have any real ... symptoms that you can say, ‘This is the cause my kidneys aren’t functioning’.” CKD 3, 70+, male, white
- “I think sometimes it’s not explained very well … what it means, especially what it means to the future …” CKD 5, 50 – 70 years, female, white
- “You don’t want this disease any more than say you want heart failure or something so I think at least I can speak for myself that the … that’s the biggest obstacle I’ve been in …” CKD 5, 50 – 70 years, male, white

**Facilitators**

- “… I just think it is just something that, that’s something that you gotta get used to. I don’t think that, you know, all the talking, you know. It’s still going to take for you to get used to that and get prepared for that. And I think if you’re really serious about living, I think it’s ... you pretty much come to reality that, okay, if this is what it takes to stay above ground, then I, I’m willing to do anything it takes. And I think when you get to that point, I think, you know, you’ll start feeling better about it. You know, and understand it.” CKD 5, 50 – 70 years, female, black

### Perceptions of terminology (91 total statements) 

Patients were asked about terms used to describe their diagnosis (i.e., “chronic kidney disease”). Follow-up probes included asking whether they’d suggest other terms to describe their condition. Patients were split with nearly half of participants who thought “chronic kidney disease” was appropriate and the other 50% who thought other terms should be used. Reasons for using other terms included a lack of understanding about the word “chronic” and the ‘harsh’ sound of the word “disease”. There were few suggestions on alternative wording that would be better.

- “… when they say ‘chronic’ it is critical I thought of and it’s mindboggling to know that. … I don’t think there’s no way of solving that. I mean … you can use the term different but it … I think the chronic has more grab (of) your attention … like you got to take care of yourself.” CKD 3, 50 – 70 years, female, black
- “Chronic meaning it’s been lasting for a long time. So I think it’s okay … This is real life here, you know, so why change (laugh) what don’t need to be changed.“ CKD 5, 30 – 50 years, male, black
- “No, I think you ought to be honest about it … and those, those words are absolutely correct. It is chronic and it is … it is a disease, you know?” CKD 3, 70+ years, female, white
- “… I think chronic anything, geriatric is another one. Those terms, you should not refer to old people as geriatric because there are the bad connotations. I think there are also bad connotations with chronic.” CKD 3, 50 – 70 years, female, white
- “It’s confusing … Not knowing … the definition of the word chronic …” CKD 4, 50 – 70 years, female, multi-race
- “I think it’s fine. It’s chronic, it’s kidney disease. In fact I think there is a tendency for some doctors, specifically earlier before I, you know, had it for a while and everything but, who would refer to it as insufficient ahh whatever, insufficient … it was kind of a, made it sound like, you know, you’re low on a vitamin or something …” CKD 3, 70+ years, male, white
- “It is a severe sounding thing but once it’s explained, in my case, it just doesn’t make a lot of difference …” CKD 3, 50 – 70 years, female, white
- “It sounds so final. It makes it seem like, you know, it’s chronic. That you, you know, maybe they need to come up with a better word because it do that. It makes you feel like it’s ... if you’re standing at the cliff and that you about ready to jump and go over the cliff …” CKD 5, 50 – 70 years, female, black

## Discussion 

Using semi-structured interviews, we found patients want to know about their CKD diagnosis and want this information early. Although hearing the diagnosis instills fear of dialysis and the “unknown”, patients indicated that these fears can be alleviated with more explanation on terminology and placing CKD in proper context to overall health. Patients consistently emphasized a desire to change behaviors early in the disease process. However, it was notable that fewer statements were offered about what behaviors patients actually did change as compared to more statements on their emotional reaction. Barriers to understanding included lack of clear explanation and low prior familiarity with CKD. 

These findings are important and in many ways novel. Contrary to prior research with providers [4], patients described a strong desire to know about a CKD diagnosis early in disease, even if it would not change clinical management. In fact, frustration occurred when some patients perceived communication about their diagnosis to be late – or as in one example, through insurance denial. Similar to focus groups of patients with advanced CKD, we found lack of early communication about diagnosis was perceived by some as withholding of information and provider paternalism [5]. 

Although there is a paucity of research examining the impact of increasing early CKD diagnosis awareness, research in diabetes has established patient awareness of diagnosis as central to education and behavior change. Diabetes education programs show improvement in glycemic control and outcomes, especially in patients most vulnerable [23]. Education to prevent diabetes shows similar promise. A recent trial of a lifestyle intervention called the “Fit Body and Soul” program was adapted from the Diabetes Prevention Program to focus on health education through community churches in pre-diabetic Blacks [24]. After 12 weeks participants receiving the intervention showed significant reductions in fasting plasma glucose and overall weight compared to those not receiving the intervention. These differences became larger after 12 months. This tells us early disease education in chronic conditions can benefit outcomes and may even ameliorate full manifestation of a disease. 

Patients pointed out in our study that there are barriers to effective diagnosis messaging beyond the patient-provider interface. Lack of symptoms, lack of general awareness about kidney disease and patient denial were all barriers cited. Clearly patients highlighted a need to address emotions of fear that come with a CKD diagnosis – not by avoiding messaging, but by talking about CKD early. Not surprisingly, studies in other chronic conditions show patient stress does increase when learning about diagnoses [25]. Research by Henry et al. [26] suggests there is an opportunity for providers to help with this by taking time during initial visits to focus on discussing diagnosis information and waiting to discuss management/treatment only after emotional aspects are addressed. Otherwise, patients may not be equipped to move from learning that they have a disease to acting on behaviors to manage it. Best outcomes may be stymied as a result. 

Patient perceptions of CKD terminology were mixed. Some felt the term “chronic kidney disease” was appropriate, and others thought this terminology was overwhelming – even describing it as having a “bad connotation”, “like geriatric”. Our goal in asking patients to comment on verbiage was meant to shed insight into how terminology may impact patient perception and acceptance of disease. Opinions were divided about terminology but there appeared unity from patients in that whatever terms are used, they should be consistent and clearly defined for patients. This is especially poignant given past and current debate from the nephrology community on when a CKD diagnosis truly is a diagnosis vs. when it is perhaps dubious to even use the term [27]. 

There are some limitations inherent to this study. Most participants were white and had high education attainment. As a result, it is difficult to surmise preferences in all patients. There may have been potential for this to limit emergence of new themes brought forth in a more heterogeneous population. However, our study provides a unique opportunity to highlight perspectives about diagnosis messaging from a sample similar to those with CKD across the U.S. Moreover, it is one of the largest studies we are aware of using patient insights from individualized interviews that encourage full participation from each participant – as compared to focus groups, whereby some individuals may be less apt to voice input within a group. Also, qualitative interpretation may be subject to potential influences of prior work of the research team. To minimize the potential of this we used two quality improvement methods to systematically develop questions used in the interviews. In addition, the study team included many who had never worked in the area of kidney disease and less likely to have preconceptions about this research. It would have been interesting to examine whether patients would want to know a CKD diagnosis even if they fully understood that they were at very low risk for progressing to ESRD. However, we feel this concept was addressed to a good extent by asking whether they would want to know even if their doctor did not think it would change management, and also, by including interview participants at early stages of CKD. The fact that patients were enrolled from a nephrology practice likely means they have more severe disease than those seen in primary care. They were also likely to have been given information about their diagnosis prior to seeing a nephrologist, although prior work shows up to 30% may still not know they have CKD even if established under care of a kidney specialist [2]. As such, patients with even less knowledge of their diagnosis may have additional perspectives that we did not capture. Lastly, after analyzing transcripts, we did not go back to participants and confirm whether themes resonated with them. Our study was not designed for follow-up cognitive interviewing but this could be considered in future work. 

Despite the limitations, there are important implications of this research. Consistent with prior research a large proportion of our participants (49%) reported they did not know why they had CKD [28]. One patient described feeling that nothing could be done to change the course of CKD. Thus, there is a continued opportunity even in nephrology care to clarify key information related to diagnosis and its management for patients. Despite fears about having CKD, patients do want to be informed about it and want this information at the earliest point in disease. This implies that providers must unify on when CKD is relevant as a disease discuss with patients. It also suggests a critical unmet need to deliver diagnostic information consistently in terms that “tell it like it is” but in a manner that recognizes and addresses the emotions that come with hearing “bad news”. 

There are many barriers patients face to understanding their diagnosis but perhaps one of the biggest opportunities for future educational research is to examine ways to initially present a CKD diagnosis to patients. Once we better understand an optimal process for delivering the diagnosis we will be better equipped to focus on next steps of management in patient-centered care. 

## Funding 

This work was supported by an NIH NIDDK K23-DK097183 (JWN). 

## Conflicts of interest 

There are no conflicts of interest to report. 

The opinions expressed are the authors and do not reflect those of the Department of Veterans Affairs. 

## Prior reporting 

A portion of the data presented in this manuscript was presented as a poster at the American Society of Nephrology 2015 Conference in San Diego, CA, USA. 

**Table 1.** Topic areas of interview questions based on cause/effect analysis and quality function deployment.

Quality improvement method	Topic areas for questions
Cause and effect analysis: “Effects”	Cause and effect analysis: “Causes” (patient, provider, system, environment)
Low patient CKD knowledge Non-optimal shared decision making Patient management/treatment decision conflict Non-optimal patient self-care, engagement Kidney disease complications, progression	What are causes for low patient disease knowledge? Where are self-care information needs unmet? What are barriers to shared decision making? What decisions do patients face about treatment, management? What factors influence them? What areas of management/treatment are difficult for patients? What are influencing factors in management decisions and daily self-care? What are causes to non-optimal patient self-care, and engagement?
Quality function deployment	Attributes of decision aid and relative importance
Preferences, needs, and desires to support patient education, decision making, patient self-efficacy, self-care, and disease management	What information is most important to know about CKD diagnosis? What information is most important to support patient self-care and management decisions? What attributes are most important in format, delivery and implementation of decision aids? What requirements (resources, needs, desires) do providers/patients have for using a decision aid practice?

**Table 2.** Baseline participant characteristics, self-reported.

Characteristic (N = 49)	Mean (SD) or n (%)
Age (years)	62 (14)
Female	25 (51%)
Race*	
White	39 (81%)
Black	5 (10%)
Asian	2 (4%)
Middle-Eastern	1 (2%)
Multi-race	1 (2%)
Annual household income^+^	
< $25,000	12 (27%)
$25,000 – 50,000	10 (22%)
≥ $50,000	23 (51%)
Formal education/highest number of years completed	
≤ H.S. diploma	13 (27%)
Some college	15 (31%)
Bachelor degree	11 (22%)
Post-graduate	10 (20%)
Partnered or married	28 (58%)
eGFR mL/min/1.73m^2^ (from records, closest to visit)	1 (8%)
> 60	6 (12%)
45 – 59	6 (12%)
30 – 44	16 (33%)
15 – 29	17 (35%)
< 15	4 (8%)
Knew cause of CKD^#^	25 (51%)
Diabetes	8 (32%)
High blood pressure	8 (32%)
Cystic disease	4 (16%)
Other (included glomerulonephritis, infection, age, medication, stones)	11 (44%)

*n = 48 reported; +n = 45 reported; ^#^patient may have listed more than one cause.
